# Familiarity expands space and contracts time

**DOI:** 10.1002/hipo.22672

**Published:** 2016-11-02

**Authors:** Anna Jafarpour, Hugo Spiers

**Affiliations:** ^1^ Helen Wills Neuroscience Institute and Psychology DepartmentUniversity of CaliforniaBerkeleyCalifornia; ^2^ Division of Psychology and Language Sciences, Department of Experimental Psychology, University College LondonInstitute of Behavioural NeuroscienceLondonUnited Kingdom

**Keywords:** human spatial navigation, grid cells, time cells, sketch‐maps, time estimation

## Abstract

When humans draw maps, or make judgments about travel‐time, their responses are rarely accurate and are often systematically distorted. Distortion effects on estimating time to arrival and the scale of sketch‐maps reveal the nature of mental representation of time and space. Inspired by data from rodent entorhinal grid cells, we predicted that familiarity to an environment would distort representations of the space by expanding the size of it. We also hypothesized that travel‐time estimation would be distorted in the same direction as space‐size, if time and space rely on the same cognitive map. We asked international students, who had lived at a college in London for 9 months, to sketch a south‐up map of their college district, estimate travel‐time to destinations within the area, and mark their everyday walking routes. We found that while estimates for sketched space were expanded with familiarity, estimates of the time to travel through the space were contracted with familiarity. Thus, we found dissociable responses to familiarity in representations of time and space. © 2016 The Authors Hippocampus Published by Wiley Periodicals, Inc.

Spatial information is often communicated by estimating time to arrival (ETA) or by sketching maps; but people are often not accurate. Sketch‐maps are often distorted, incomplete, and/or do not align with a scaled map (Kuipers, 1982). Studies on sketch‐maps revealed insights into how the internal representation of space (cognitive maps) relates to the real‐world (Kosslyn et al., 1974; Taylor and Tversky, 1992; Denis et al., 2014). For instance, the accuracy of sketch‐maps shows the precision of the cognitive maps (Golledge et al., 1992). The precision of cognitive maps is also revealed by ETA (Yamamoto et al., 2014). Yet, the link between spatial and temporal aspects of cognitive maps is not clear. Here, we hypothesized that, if both spatial and temporal inferences are driven from the same cognitive map, the distortion should similarly affect sketch‐maps and travel‐time estimations. But if the temporal and spatial aspects of cognitive map are represented or processed separately, distortions on temporal and spatial expressions may dissociate.

Generally, ETA is proportional to the distance to the destination (Golledge and Zannaras, 1971; Plumert et al., 2005); but, a number of factors can alter the perception of ETA, such as the emotions during traveling (Downs and Stea, 1973), attention (Ozawa et al., 2015), path direction (Säisä et al., 1986; Hanyu and Itsukushima, 1995) and familiarity with the space (Säisä et al., 1986; van de Ven et al., 2011; Ozawa et al., 2015). In this study, we focused on the impacts of familiarity with space on ETA and sketch‐maps.

Familiarity with the space leads to smaller spacing between grid peaks of the firing rates of grid cells in the entorhinal cortex of rodents (Barry et al., 2012). Given that grid‐like cells have been recorded in human entorhinal cortex (Jacobs et al., 2013), we hypothesized that familiarity with an environment would lead to an expansion in the relative size of the environment in mental representations and on sketch‐maps. Grid cells have been argued to provide an internal metric of an environment (Moser et al., 2008), with distances calculated from the number of activity traveled through peaks, when traversing the space ‐ analogous to travel across latitude and longitude lines in cartographic space (Moser et al., 2008; Bush et al., 2015; Spiers and Barry, 2015). We further hypothesized that, assuming a linear relationship between time and space, reduction in grid cells spacing (grid units) would lead to ETA for familiar paths being longer (more grid units per meter) than unfamiliar paths (less grid units per meter).

To evaluate this hypothesis, we tested young adults (*n* = 20, male/female = 13/7, mean age = 27 (SD = 3)) who had been living in the same building (William Goodenough House, *Bloomsbury*, London WC1N 2AB, United Kingdom) for nine months, with no prior knowledge of the area, and had been traveling in that area on foot only (no cycling or driving). All participants gave written informed consent. They were financially compensated for their participation. And the University of London Research Ethics Committee for Human‐based Research approved the study.

The experiment was conducted on the ground floor of William Goodenough House where participants sat facing the south wall toward the exit of the building. All drawings were on A4 size white paper. None of the tests were time‐limited and participants stopped sketching whenever they wanted. The experiment had multiple stages: first, we administered the Rey‐Osterrieth Complex figure test to familiarize participants with the experimental setup. Then, participants were asked: “*Please draw a map of Bloomsbury as it comes to your mind*.,” “*Draw whatever comes to your mind*.,” and “*The area is as big as it can fit on the page, given the probe scale*.” We then explained what the probe was (Fig. 1). Our instruction proscribed participants from intentionally exaggerating or squeezing in drawings. We used a satellite image of the Bloomsbury (from maps.google.com) in 200 m in 1 inch scale as our guideline. That scale comfortably fits in an A4 size page. The scale of sketch‐map was probed by a square on an empty A4 page resembling the outline of the William Goodenough building (Fig. 1). Participants were instructed to draw the map with south along the top of a page; the same direction as they were facing during the experiment. We used this direction to avoid drawings based on visual memory of the map of the Bloomsbury district.

**Figure 1:**
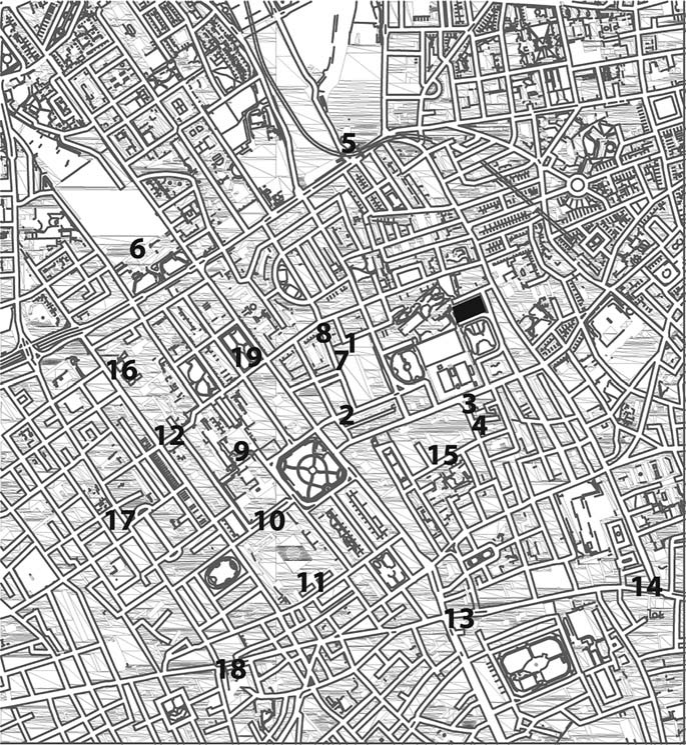
Map of the Bloomsbury district roads and paths. William Goodenough House is marked in solid black – its outline probed the scale for sketching maps (note: this figure is not the probed scale). We asked ETA to 19 destinations which are within about 1 mile from the William Goodenough House. They are marked by numbers. (1) Waitrose, (2) Russell Square tube station, (3) the Lamb pub, (4) People's supermarket, (5) Kings Cross tube station, (6) Euston tube station, (7) Boots, (8) Marchmont street, (9) SOAS, (10) front door of the British Museum, (11) back door of the British Museum, (12) Waterstones, (13) Holborn tube station, (14) Chancery Lane tube station, (15) Great Ormond Street, (16) UCL main entrance, (17) Goodge Street tube station, (18) Tottenham Court Road tube station, (19) Tavistock Square.

**Figure 2:**
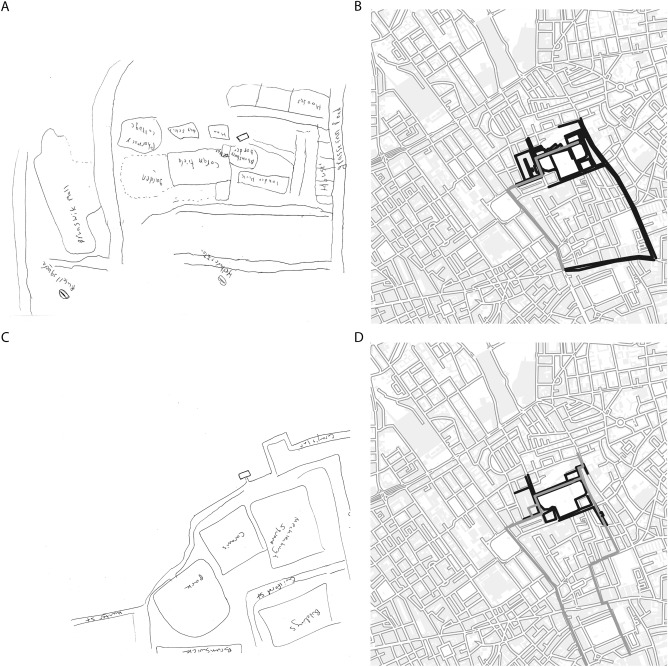
(A and C) two examples of sketch‐maps. (B and D) Dark gray shows what has been covered on the examples (A) and (C) respectively. And light gray shows the participants' daily visited routes. (A to D) North is up. Note that the sketch‐maps were drawn south up.

After drawing, the sketch‐map was taken away; participants answered to “*How long does it take, in minutes, to walk from the William Goodenough House to (questioned destinations)?*” (See the 19 destinations in Fig. 1). If they did not know the destination, we discarded the entry (2.4 (SD = 2.2) out of 19 entries were discarded]. Finally, a satellite map of the Bloomsbury district was given to the participant to mark at least 2 to maximum 5 routes within the area that they took most frequently, “*on daily basis*” (Fig. 2).

We found that ETA correlated with actual distance in the real world (within participant: mean = 0.807, SD = 0.1; across participants: t(17) = 33.75, *P* < 0.001). The distance was measured as “path distance” (Howard et al., 2014). The ETA error was the difference between participants' estimations and data from maps.google.com (which is based on walking in normal constant speed, without breaks, but considering changes to speed with respect environmental conditions such as slope). The ETA errors did not increase or decrease with the distance to the destinations (r = −0.064, SD = 0.395; t(17) = 0.69, *P* = 0.499).

We focused on the length of paths in the sketch‐maps, based on the approach of prior studies (Golledge and Zannaras, 1971; Plumert et al., 2005). Every continuous drawn line depicting a street or a way to travel was counted as a path. Paths along the daily visited routes were categorized as the “highly‐familiar paths” and other paths were the “less‐familiar paths.” Two participants' data were discarded from the analysis. One of them was discarded because the participant did not draw any paths, and the other one was discarded because all paths were indicated as daily walking routes. We also discarded any path which was drawn (at least in part) within 1 inch from edges of the A4 page. This was to avoid including any drawing which may be in smaller scale just to be squeezed onto the page (1.65, SD = 1.79 paths per drawing were discarded; 45% of participants did not draw close to edges; 41.3% of discarded paths were the highly‐familiar ones). Next, we measured the precision of paths sketches (length of drawn path relative to the scale of the building displayed on the paper provided). The drawings in the same scale were considered accurate (drawing scale/cue scale = 1).

On average, participants drew 6.8 paths (SD = 4.59), of which 4.5 (SD = 2.68) were highly‐familiar and 2.3 (SD = 2.54) were less‐familiar (Fig. 3A). Both highly‐familiar paths (3.15, SD = 1.55) and less‐familiar paths were drawn longer (2.26, SD = 1.39) than the probe scale. That may be because all sketched paths were familiar to the participant but some paths were more familiar than others. The expansions correlated (*r* = 0.567, *P* = 0.0091). But highly‐familiar paths were drawn significantly longer than less‐familiar paths (*t*(17) = 2.975, *P* = 0.0085; Fig. 3A).

**Figure 3:**
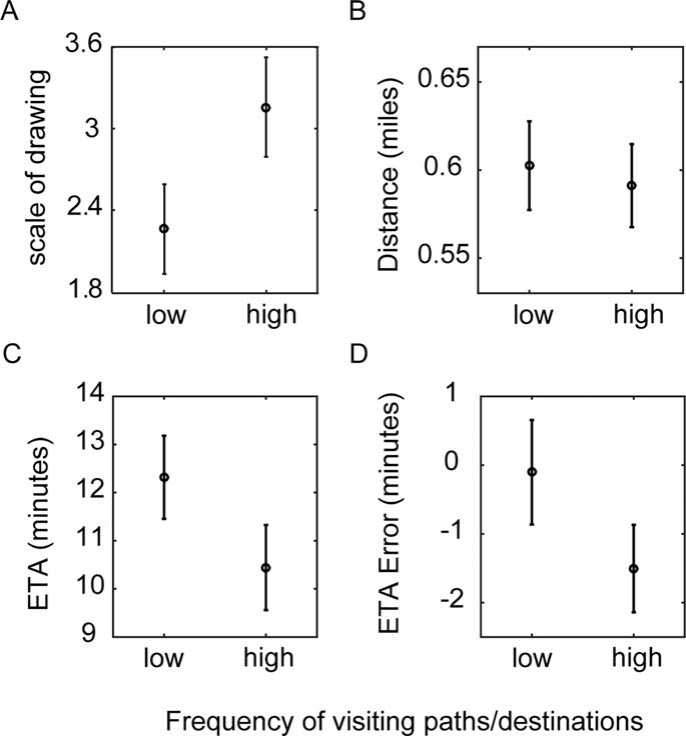
(A) The averaged scale of drawn paths: highly‐familiar paths (high) were drawn longer than less‐familiar paths (low) (*P* < 0.05). (B) The averaged path distance for selected high and low frequently visited destinations (*P* > 0.25). (C) The averaged estimated time of arrival (ETA) for frequently visited destinations (high) was less than less‐frequently (low) visited destinations (*P* < 0.05). (D) The averaged ETA error shows underestimation of travel‐time to highly‐familiar destination (*P* < 0.05). In all panels, error‐bars show SEM (*n* = 18 participants).

We tested ETA to destinations with matched distances. Destinations on the daily visited paths were considered as highly‐familiar, and other familiar destinations were less‐familiar. Participants did not necessarily draw the questioned landmarks in their sketch‐maps. The highly‐familiar destinations were usually closer to the college than less visited destinations (averaged distance to highly‐familiar destinations: 0.46 miles SD = 0.04, and less‐familiar destinations: 0.53 miles SD = 0.1). Therefore, for each participant, we stratified samples to control for the distance effects: the ranges of distances between the building and destinations were set to be the same for highly‐familiar and less‐familiar groups (by excluding highly‐familiar destinations which were closer to the building than the closest less‐familiar destinations and excluding less‐familiar destinations which were further away than the farthest highly‐familiar destination). After this matching, there was no difference in the number of turns (*t*(17) = 0.43, *P* = 0.66), or the distance (along the path *t*(17) = 0.53, *P* = 0.60, Euclidean *t*(17) = 1.3, *P* = 0.21, “Euclidian distances” and “path distances” were highly correlated, *r* = 0.967, *P* < 0.001; Fig. 3B).

If space and time estimates were consistently distorted in memory, we would predict that ETA for highly‐familiar destinations would also expand, given our sketch‐map results. However, we found the opposite of this. The average ETA for highly‐familiar destinations was shorter than the average ETA for less‐familiar destinations (*t*(17) = 2.17, *P* = 0.044; Fig. 3C). We next measured the accuracy of ETA for highly‐familiar and less‐familiar destinations relative to maps.google.com estimates. We found that ETA for highly‐familiar destinations was underestimated (−1.5, SD = 2.69) in comparison to less‐familiar destinations (−0.1, SD = 3.22; Fig. 3D).

In summary, while estimates for sketched space expanded with familiarity, estimates of the time to travel through the space contracted with familiarity. Past research exploring impacts of familiarity on sketch‐maps (10 exposures over 10 weeks) found no changes over time in the accuracy of the sketch‐maps for the parts of the environment directly traveled through (Ishikawa and Montello, 2006). Notably, our experiment had several differences which may explain why we found an effect of familiarity: as such, our participants had longer exposure to the environment (9 months of living in the environment), we compared the paths drawn for regions judged as highly‐familiar and less‐familiar, rather than sampling the environment as a whole, and we instructed sketching maps to be south‐up to avoid sketching based on visual memories of maps.

Although our ETA results were not consistent with the initial hypothesis, they were consistent with previous reports on underestimating travel‐time with familiarity to newly encountered environments (Seno et al., 2011; van de Ven et al., 2011; Ozawa et al., 2015). Here, we tested memory for the space after 9 month of exposure. Paths to highly familiar destinations may schematized over time and remembering them required less details and retrieval demands than newly encountered paths (Hirshhorn et al., 2012). In fact, spatial familiarity leads to fast and vivid recall of scenes and episodes (Robin and Moscovitch, 2014; Herdman et al., 2015; Arnold et al., 2016). Thus, the difference in retrieval demands may explain the ETA contraction with familiarity.

What neural mechanisms may underlie the dissociable effects of familiarity on time and space? We hypothesized that expansions of space, as seen in the sketch‐maps, were a consequence of the expansion in grid spacing, as observed in rodent grid cells (Barry et al., 2012). Current theories argue that a unified representation of space in hippocampal‐parahippocampal regions characterizes the environment for navigation and memory (Moser et al., 2008; Bush et al., 2015; Spiers and Barry, 2015; Horner et al., 2016). In this framework, drawing a path or estimating time to reach a location should result in matching estimates (distorted with a similar bias). Our data showed this is not the case. Thus, separate neural systems may represent spatial and temporal information, or a single neural system may represent both space and time but it is processed differently for temporal and spatial estimates.

Taking the “separate systems” approach, one account is that temporal lobe structures store the semantic knowledge about the average travel‐time to commonly traveled destinations (Patterson et al., 2007); for example, it takes 5 min to walk to the local subway. By contrast, there may be no semantic knowledge of what the “south‐up” sketch‐map should look like; therefore, it draws on a separate system for recall of the spatial details. An alternative account is that both spatial and temporal representations of the environment are stored separately in hippocampal‐parahippocampal regions. Distances might be represented by entorhinal grid cells and hippocampal “place cells” (which express activity in localized regions of an environment), and travel‐times encoded by hippocampal “time cells” (which encode time elapsed; MacDonald et al., 2011; Eichenbaum, 2014; Kraus et al., 2015). Broadly consistent with this, the parahippocampal regions are more active during spatial recall than during temporal recall, and the hippocampal regions more active during temporal recall than during spatial recall (Ekstrom and Bookheimer, 2007)—suggesting separate systems. In this account, time cells may adjust their firing patterns with increasing exposures to the environment, such that readout from the time cells leads to underestimates in ETA to highly‐familiar destinations.

An alternative “differential processing” account is that, for sketch‐maps, vectors between the landmarks and buildings may be retrieved from the hippocampal‐parahippocampal system. Such vector readout might depend directly on the grid‐cell representations, which leads to overestimating familiar regions in sketch‐maps. ETA would involve retrieval of the same long‐term memory store, but via a different mechanism: involving replay of sequences of hippocampal place cells along the routes. Familiarity may increase efficiency of the replay, which may underlie the fast retrieval (Robin and Moscovitch, 2014) and trigger the travel‐time underestimation to highly‐familiar destinations. Indeed, hippocampal replay like responses has been evident in humans (Ritchey et al., 2013; Jafarpour et al., 2014; Horner et al., 2015), and it is possible that the time taken to recall a path compresses, similar to the temporal compression shown in rodents (Bonasia et al., 2016).

In conclusion, we found dissociation between effects of familiarity on the spatial and temporal estimations of an environment, which we suggest may relate to differences in temporal and spatial tuning of cognitive maps or the speed of accessing source memories. Future studies would be useful to explore individual differences and neuroimaging data would be informative to assess potential mechanisms. It will be worthwhile to determine whether hippocampal activity which correlates with the distance to goal locations (Sherrill et al., 2013; Howard et al., 2014; Spiers and Barry, 2015) is expanded or contracted by familiarity; and whether hippocampal time cell coding also changes with familiarity.
